# Aneurysm of the Left Coronary Artery in Postoperative Bland-White-Garland Syndrome

**DOI:** 10.1155/2015/568014

**Published:** 2015-12-06

**Authors:** Nathalie Jeanne Magioli Bravo-Valenzuela, Guilherme Ricardo Nunes Silva

**Affiliations:** ^1^Pediatrics Department, University of Taubaté, 12020-130 Taubaté, SP, Brazil; ^2^University of Taubaté, 12020-130 Taubaté, SP, Brazil

## Abstract

We report a case of anomalous left coronary artery from the pulmonary artery (ALCAPA) or Bland-White-Garland syndrome, present the challenges of performing a differential diagnosis, and discuss the treatment of the syndrome. Although ALCAPA is a rare congenital heart disease, it is one of the most common causes of myocardial ischemia in childhood and presents a diagnostic challenge. A four-year-old girl was referred to a pediatric cardiologist for evaluation of mitral valve regurgitation murmur and heart failure. The transthoracic echocardiogram demonstrated the left coronary artery (LCA) not arising from the aorta, presence of coronary collateral circulation, and moderate mitral valve regurgitation. ALCAPA was confirmed using angiotomography. The LCA was surgically reimplanted into the aorta. After 3 years of postoperative follow-up, the patient developed an LCA aneurysm. Diagnosis of cardiac ischemia in childhood remains a challenge, and careful evaluation of coronary arteries on the echocardiogram is an important tool. In this report, we present a case of ALCAPA with an uncommon postoperative outcome.

## 1. Introduction

Anomalous left coronary artery from the pulmonary artery (ALCAPA) or Bland-White-Garland syndrome is a rare congenital heart disease accounting for 0.25–0.5% of all congenital heart diseases [1]. It is one of the most common causes of myocardial ischemia in childhood and, if left untreated, it is potentially fatal with a mortality rate of 80–90% within the first year of life [2].

The most common clinical presentation of ALCAPA includes paroxysm of crying, pallor, diaphoresis, and signs of heart failure in infants. Diagnosis of myocardial ischemia in childhood presents a challenge and is commonly misdiagnosed as colic in infants. In cases in which cardiac ischemia does not develop into a lethal event, it can cause dilated cardiomyopathy, heart failure, and/or mitral valve regurgitation, as in the case reported here. In 10–15% of cases, patients remain asymptomatic until a sudden lethal event occurs after intense physical activity, as a result of cardiac ischemia. The clinical course depends on the degree of collateral vessels between the coronaries, which allows patients to survive till adulthood [3].

Dual coronary repair by direct aortic reimplantation is the treatment of choice for ALCAPA, and surgery should be performed as soon as the diagnosis is confirmed. Long-term results are satisfactory and the risk of late stenosis or occlusion of the reimplanted coronary is lower. However, the development of left coronary artery (LCA) aneurysm after surgical correction of ALCAPA is extremely rare [4].

This is a case report of a pediatric patient who presented two years after repaired ALCAPA (direct reimplantation of the LCA into the aorta) with an aneurysm of the LCA diagnosed on echocardiography, focusing on the difficulties of this diagnosis.

## 2. Case Presentation

A four-year-old girl was referred to a pediatric cardiologist due to low weight and heart murmur. She was born 38 weeks into gestation by cesarean section and weighed 2.915 g, with an Apgar score of 9 at 1 min as well as at 5 min. During the first 2-3 months after birth, the parents related that she experienced episodes of pallor and irritability, which were diagnosed as infant colic.

At first evaluation, the child was acyanotic and weighed less for her age (*Z*-score = −2) based on WHO growth charts and presented symptoms of exercise intolerance. The heart rate was 129 beats/min, and blood pressure was 90/60 mmHg. Cardiac auscultation showed normal heart sounds (S1 and S2) and a systolic murmur (grade 3/6) at mitral area with radiation to the axilla.

**Figure 1:**
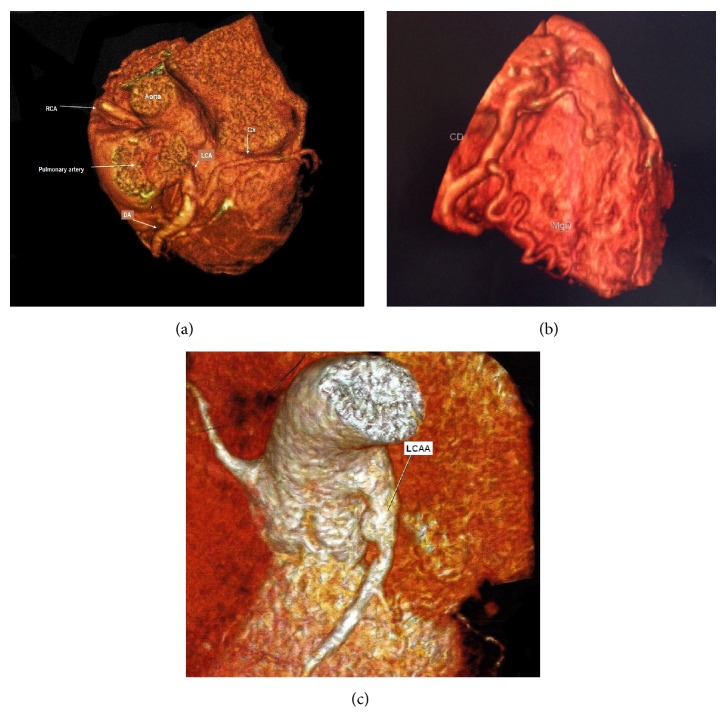
(a) Computed tomography (CT) angiography showing the left coronary artery (LC) from the pulmonary trunk (RCA: right coronary artery; AD: anterior descending artery; and Cx: circumflex artery). (b) CT angiotomography showing the coronary collateral network. (c) CT angiotomography showing the left coronary artery aneurysm.

The initial electrocardiogram (ECG) showed sinus rhythm at 135 beats/min, normal QTc (0.42 s), signs of left ventricular enlargement, negative and deeply inverted T waves at V4-5-6, with normal Q waves. Chest radiography demonstrated cardiomegaly. Transthoracic echocardiogram (TTE) revealed left ventricular dilatation with normal systolic function, moderate mitral valve regurgitation, flow from the LCA to the pulmonary trunk on color Doppler, and diffuse ectasia of right coronary artery (RCA) with coronary collateral circulation. ALCAPA was confirmed by coronary computed tomography (CT) angiography (Figures 1(a) and 1(b)).

The child was successfully operated on with direct reimplantation of the LCA into the aorta. The ostium of the LCA was excised with a button of surrounding pulmonary artery tissue, and the pulmonary artery was transected. The LCA was well mobilized to the left side of the aortic root, ensuring no tension or kinking of the artery. The defect in the pulmonary artery was repaired with a pericardial patch. Postoperative TTE showed LCE connected to the aorta with normal flow, mild mitral valve regurgitation, and normal left ventricle ejection fraction (Simpson's method). After a period of 6 months, the antiplatelet drug (aspirin) was discontinued. After a 3-year follow-up, the patient presented an episode of chest pain during physical activity. ECG showed T waves at V4-5-6 and normal troponin T levels (0.06; normal < 0.014). TTE revealed the presence of a saccular aneurysm of LCA (6.5 × 8 mm/0.4 cm^2^). A coronary CT angiography showed no stenosis or thrombus and a coronary aneurysm type IV at the point of LCA-aorta anastomosis (Figure 1(c)). Tests for possible systemic diseases related to the coronary aneurysm were negative, showing normal blood cells, normal erythrocyte sedimentation rate, normal PTT, anti-DNA and anticardiolipin negatives, and normal levels of C3, C4, CH50, and C-reactive protein. Screenings for atherosclerotic diseases and genetic syndromes were also negative. In order to evaluate cardiac perfusion, a rest-exercise stress myocardial scintigraphy was obtained, which was also normal. The patient is currently asymptomatic and is taking aspirin and a beta-blocker. High-impact, competitive, and collision sports were prohibited and stress tests have been performed annually to guide recommendations for physical activities.

## 3. Discussion

ALCAPA or Bland-White-Garland syndrome is an extremely rare congenital heart disease with an estimated occurrence of 1 in 300.000 live births. Although it is generally an isolated defect, approximately 5% of the cases can be associated with other cardiac anomalies [5]. Similar to colic in infants, the myocardial ischemia may present as paroxysms of pallor and irritability and should be suspected when those signs are associated with heart failure, ECG changes, and cardiomegaly [3].

In pediatric patients, the TTE is considered as the main noninvasive diagnostic method of investigation [6]. Real-time 3D echocardiography represents a new approach to improve the cardiac images in the conventional TTE. Diagnostic criteria for ALCAPA using echocardiography include: a dilated RCA, a reversed color Doppler flow from the LCA to the pulmonary artery, and a prominent septal flow from collateralization. The diagnosis must be confirmed by identifying the origin of the LCA from the pulmonary trunk [3]. Other noninvasive methods include CT or magnetic resonance coronary angiography with excellent accuracy in assessing the origin of LCA anomalies [7]. Cardiac catheterization should be indicated to establish the diagnosis when other noninvasive methods fail. Furthermore, myocardial scintigraphy is the preferred method to approach myocardial viability and risk stratification in coronary artery disease. Currently, most stress myocardial scintigraphy is performed using ECG-gated single-photon emission computed tomography (SPECT) for simultaneous evaluation of myocardial perfusion and cardiac function [5, 7].

Once ALCAPA is diagnosed, early surgical treatment is critical to correct the defect and prevent complications. The preferred surgical method is restoring the dual coronary system [8]. In children, surgical repair of choice is reimplantation with translocation of the LCA from the pulmonary artery to the aorta. During ALCAPA repair, the LCA must be well mobilized to avoid tension or kinking of the coronary artery and prevent postoperative complications, such as stenosis or occlusion of the reimplanted coronary and cardiac ischemia. If direct reimplantation of the LCA is not possible, it can be connected to the aorta by an intrapulmonary tunnel such as the Takeuchi technique [9, 10]. Other surgical options include simple ligation of the LCA and bypasses using saphenous vein or arteries grafts. In general, the patients showed improvement in mitral valve regurgitation and left ventricular function after the surgical correction, mainly when the operation was performed under 1 year of age [10]. Although it has ever been described a case of a giant coronary artery in an adult patient after Takeuchi repair, the finding of coronary aneurysm in a reimplanted LCA for ALCAPA is extremely rare and unexpected [4].

Atherosclerotic aneurysms, Kawasaki disease, Takayasu arteritis, and coronary artery fistula are possible differential diagnoses with ALCAPA by dilatation of the coronary artery. Important aspects of each of these diseases are described in Table 1 [3].

Clinical management of coronary aneurysm consists of attempts to prevent thromboembolic complications. Long-term antiplatelet therapy is recommended for patients with small to medium (>3 mm but <6 mm) coronary artery aneurysm, as in the case reported. Adjunctive therapy with antithrombotic regimen is recommended for patients with large aneurysm (>6 mm) and patients in whom coronary artery contains multiple aneurysms [11]. Classification of coronary artery dilatation is described in Table 2.

The presence of a dilated LCA after surgical correction of ALCAPA could be related to stenosis at the anastomosis site (LCA-aorta) as a compensatory distal dilatation in order to provide flow to this area [5]. However, in the present case, the aneurysm of the LCA developed without proximal stenosis after a successful surgical reimplantation repair, resulting in a rare outcome [4].

## Figures and Tables

**Table 1:** Key distinguishing features of dilated coronary arteries (LCA: left coronary artery; RCA: right coronary artery). Adapted from Peña et al. [3].

Disease	Imaging findings	Differentiating features
ALCAPA	Flow from LCA to pulmonary artery	LCA arises from the main pulmonary artery
Coronary artery dilatation related to atherosclerosis	Diffuse coronary artery dilatation	Atherosclerotic plaque
Kawasaki disease	Multiple coronary artery aneurysms	Young children with fever and exanthema
Coronary artery-coronary sinus fistula	Tortuous coronary artery plus dilated coronary veins and sinus	Arteriovenous communication
Takayasu arteritis	Coronary artery aneurysms and stenosis	Involves the aorta and great vessels

**Table 2:** Group and type of dilatation of coronary arteries.

Type and group	Findings
Saccular aneurysm	Transverse > longitudinal diameter
Fusiform aneurysm	Longitudinal < transverse diameter

Type	
I	Diffuse ectasia in 2 or 3 vessels
II	Diffuse ectasia in 1 vessel and localized aneurysm in another
III	Diffuse ectasia in only 1 vessel
IV	Coronary aneurysm in 1 vessel

## References

[1] Bland E. F., White P. D., Garland J. (1933). Congenital anomalies of the coronary arteries: report of an unusual case associated with cardiac hypertrophy. *American Heart Journal*.

[2] Kirklin J. W., Barratt-Boyes B. G., Kirklin J. W., Barratt-Boyes B. G. (1986). Congenital anomalies of the coronary arteries. *Cardiac Surgery*.

[3] Peña E., Nguyen E. T., Merchant N., Dennie C. (2009). ALCAPA syndrome: not just a pediatric disease. *Radiographics*.

[4] Dunlay S. M., Bonnichsen C. R., Dearani J. A., Warnes C. A. (2014). Giant coronary artery aneurysm after takeuchi repair for anomalous left coronary artery from the pulmonary artery. *The American Journal of Cardiology*.

[5] Díaz-Zamudio M., Bacilio-Pérez U., Herrera-Zarza M. C. (2009). Coronary artery aneurysms and ectasia: role of coronary CT angiography. *Radiographics*.

[6] Ropers D., Moshage W., Daniel W. G., Jessl J., Gottwik M., Achenbach S. (2001). Visualization of coronary artery anomalies and their anatomic course by contrast-enhanced electron beam tomography and three-dimensional reconstruction. *The American Journal of Cardiology*.

[7] Cohen M. S., Herlong R. J., Silverman N. H. (2010). Echocardiographic imaging of anomalous origin of the coronary arteries.. *Cardiology in the Young*.

[8] Bunton R., Jonas R. A., Lang P., Rein A. J., Castaneda A. R. (1987). Anomalous origin of left coronary artery from pulmonary artery. Ligation versus establishment of a two coronary artery system. *Journal of Thoracic and Cardiovascular Surgery*.

[9] Takeuchi S., Imamura H., Katsumoto K. (1979). New surgical method for repair of anomalous left coronary artery from pulmonary artery. *Journal of Thoracic and Cardiovascular Surgery*.

[10] Michielon G., Di Carlo D., Brancaccio G. (2003). Anomalous coronary artery origin from the pulmonary artery: correlation between surgical timing and left ventricular function recovery. *Annals of Thoracic Surgery*.

[11] Newburger J. W., Takahashi M., Gerber M. A. (2004). Diagnosis, treatment, and long-term management of kawasaki disease: a statement for health professionals from the Committee on Rheumatic Fever, Endocarditis and Kawasaki Disease, Council on Cardiovascular Disease in the Young, American Heart Association. *Circulation*.

